# Wall Thickness of Industrial Multi-Walled Carbon Nanotubes Is Not a Crucial Factor for Their Degradation by Sodium Hypochlorite

**DOI:** 10.3390/nano8090715

**Published:** 2018-09-12

**Authors:** Alexander G. Masyutin, Dmitry V. Bagrov, Irina I. Vlasova, Igor I. Nikishin, Dmitry V. Klinov, Ksenia A. Sychevskaya, Galina E. Onishchenko, Maria V. Erokhina

**Affiliations:** 1 Faculty of Biology, 1-12 Leninskie Gory, Lomonosov Moscow State University, Moscow 119991, Russia; ii.nikishin@physics.msu.ru (I.I.N.); galina22@mail.ru (G.E.O.); erokhinam@bk.ru (M.V.E.); 2 Federal Research and Clinical Center of Physical-Chemical Medicine of Federal Medical-Biological Agency, Malaya Pirogovskaya, 1a, Moscow 119435, Russia; dbagrov@gmail.com (D.V.B.); irina.vlasova@yahoo.com (I.I.V.); klinov.dmitry@mail.ru (D.V.K.); 3 Faculty of Fundamental Medicine, 31-5 Lomonosovsky Prospekt, Lomonosov Moscow State University, Moscow 117192, Russia; ksesha1812@yandex.ru

**Keywords:** Industrial-grade MWCNTs, biodegradation, sodium hypochlorite, oxidation

## Abstract

The propensity of multi-walled carbon nanotubes (MWCNTs) for biodegradation is important for their safe use in medical and technological applications. Here, we compared the oxidative degradation of two samples of industrial-grade MWCNTs—we called them MWCNT-d and MWCNT-t—upon their treatment with sodium hypochlorite (NaOCl). The MWCNTs had a similar inner diameter but they differed about 2-fold in the outer diameter. Electron microscopy combined with morphometric analysis revealed the different degradation of the two types of MWCNTs after their incubation with NaOCl—the thicker MWCNT-d were damaged more significantly than the thinner MWCNT-t. The both types of MWCNTs degraded at the inner side, but only MWCNT-d lost a significant number of the outer graphitic layers. Raman spectroscopy demonstrated that both MWCNTs had a similar high defectiveness. Using energy-dispersive X-ray spectroscopy, we have shown that the more degradable MWCNT-d contained the same level of oxygen as MWCNT-t, but more metal impurities. The obtained results suggest that the biodegradability of MWCNTs depends not only on the wall thickness but also on the defects and impurities. Thus, the biodegradability can be regulated by the synthesis conditions or the post-synthesis modifications. Such degradation flexibility may be important for both medical and industrial applications.

## 1. Introduction

The significant advances in the applications of carbon nanotubes (CNTs) in different fields of technology and industry raise an issue regarding their sustainable and health-safe development. People contact with industrial CNTs when working with plastics, concrete, and composite materials; all of them may contain multi-walled carbon nanotubes (MWCNTs) [1,2]. Despite the promising approaches for using MWCNTs in medicine [3,4], after undesirable entering the body, MWCNTs can cause local inflammation with the migration of phagocytes, i.e., neutrophils and macrophages, to the site of their location [5,6]. The propensity of industrial-grade MWCNTs for biodegradation and elimination from the body is of great importance for their safe use.

The biodegradation of CNTs by phagocyte-derived oxidants was first demonstrated for the carboxylated single-walled CNTs (c-SWCNTs) [7,8,9]. Upon degradation, c-SWCNTs initially became shortened, and, finally, their tubular structures completely disappeared. The destruction of c-SWCNTs was accompanied by the formation of multiple intermediate compounds with CO_2_ gas as a final product [10,11].

Hypochlorous acid (HOCl) is the major oxidant produced by activated neutrophils and macrophages to fight invading pathogens. It was shown that 10^6^ activated neutrophils are able to produce 50 nmol of NaOCl within 30 min, and a larger number of neutrophils (25–50 × 10^6^) may generate NaOCl at very high steady-state concentrations (up to 25–50 mM) [12,13]. It was demonstrated to be the most effective cellular oxidant to degrade resistant structures such as SWCNTs [14].

Due to their multilayer structure, MWСNTs were shown to be more resistant to degradation than SWCNTs; prolonged exposure to oxidants was needed for the significant degradation of MWCNTs [14,15,16]. On the other hand, compared to SWCNTs, MWCNTs are usually more defective due to the rapid non-equilibrium synthesis conditions [17]. Thus, the degradation of MWCNTs is a more complex process than the degradation of SWCNTs [18,19]. ROS (reactive oxygen species) mediated MWCNT degradation inside macrophages was demonstrated to proceed via two mechanisms: a site-specific transversal drilling process on pre-existing defects of nanotubes and a non-site-specific thinning process of the walls [20]. The perforated surface of MWCNTs is peeled off layer by layer upon oxidative biodegradation [18]. The length, aggregation state, and the existing structural defects on the sidewalls of MWCNTs determine their biodegradability potential [21].

Another way to design safer CNTs with tailored biodegradability is the chemical modification (or functionalization) of the MWCNTs’ surface. Degradation of CNTs proceeded to a high extent if they had oxygenated groups (i.e., hydroxyl, carbonyl, carboxyl) on their surface [10,15,18]. MWCNT covalent functionalization was shown to affect the rate and extent of MWCNT enzymatic degradation [22,23], and the degradation of MWCNTs by macrophage-like microglia cells both in a culture model and in in vivo experiments [24,25].

The MWCNT degradation, discussed above, was explored by the use of lab-manufactured CNTs with well-known chemical modifications. There are only a few works studying the degradation of industrial-grade MWCNTs [26]. We revealed, earlier, the alterations of the MWCNT-t structure after their treatment with murine gastric juice [27]. The present study was aimed to elucidate the biodegradation of industrial-grade MWCNTs upon their treatment with sodium hypochlorite (NaOCl), which is the sodium salt of HOCl (p*K*a = 7.46). Previously, NaOCl was used just to modify the physico-chemical properties of MWCNTs in order to improve their adsorption performance towards metal ions [28,29].

Here, we employed conventional transmission electron microscopy (TEM) and analytical electron microscopy, scanning electron microscopy (SEM), and Raman spectroscopy to compare the morphological changes occurring in the two different types of industrial-grade MWCNT after NaOCl treatment.

## 2. Materials and Methods

### 2.1. MWCNTs

In the present study, we used two samples of nanotubes. MWCNTs (Taunit) (MWCNT-t) were produced by using the chemical vapor deposition (CVD) method (NanoTechCenter Ltd., Tambov, Russia) with Ni used as a catalyst. MWCNTs (Dealtom) (Dealtom Inc., Moscow, Russia, MWCNT-d) were produced by the high-pressure disproportionation technique, employing CH_4_ in a continuous-flow gas-phase as the carbon feedstock and a catalyst precursor containing Ni particles. Intact nanotubes are black powders with a low ability to form aqueous suspensions. Due to their hydrophobic nature, MWCNTs usually form aggregates in an aqueous environment. When examined using an electron microscope, single MWCNTs are seen as hollow cylinders ((Taunit): http://eng.nanotc.ru/producrions/87-cnm-taunit, (Dealtom): https://dealtom.all.biz/en/multilayered-carbon-nanotubes-munt-dealtom-g3847601). Nanomaterials were chosen due to the similarity of their characteristics provided by the manufacturers.

### 2.2. Incubation of MWCNTs with Hypochlorite

Suspension of MWCNTs (500 µg/mL) in water was aliquoted (250 µL) into Eppendorf tubes immediately after rigorous stirring. MWCNTs were sonicated in an ultrasonic bath (Elma Ultrasonic, Singen, Germany) at 25 °C, power 90 W. Micro aliquots of freshly prepared NaOCl (Sigma-Aldrich, St. Louis, MO, USA) were added to the tubes once or twice daily over a period of 10 days. Concentrations of NaOCl were added in the samples as 1 mM (NaOCl^low^) and ~100 mM (NaOCl^high^). During the incubation period, 1 mM NaOCl was added 11 times, and 70–100 mM NaOCl added 6 times. Control MWCNTs suspensions were supplemented with an equivalent volume of water. Treatment history is presented in Table S1 in the Supplementary Information. After the last addition, the incubation was continued for 5 days more. The samples were incubated at room temperature upon shaking during several hours a day. After the treatment with NaOCl the samples were centrifuged (10,000 *g*) several times and washed with an excess amount of distilled water in order to remove any possible residual NaOCl. The experiment was conducted twice for each type of MWCNTs.

### 2.3. Electron Microscopy Analysis

For transmission electron microscopy and analytical electron microscopy, the aqueous suspension was stirred rigorously; one drop of the suspension of MWCNTs was deposited on copper grids, coated with Formvar film and dried at room temperature for 24 h. The MWCNTs were observed by an analytical electron microscope (JEM 2100) (Jeol, Tokyo, Japan) (200 kV, non-corrected, LaB6 cathode): a Gatan Orius SC200D 2k camera (Gatan, Pleasanton, CA, USA) was used. A Gatan FT1000 2k camera (Gatan, Pleasanton, CA, USA) was used for the electron diffraction method.

For the scanning electron microscopy, the aqueous suspension was stirred rigorously, then one drop of the suspension of MWCNTs was placed on a glass cover slip and dried at room temperature for 24 h. Dried specimens were sputter-coated with a thin film of gold and examined using a scanning electron microscope (JSM-6380 LA) (Jeol, Tokyo, Japan), 20 kV.

### 2.4. Morphometric Analysis

Wall thickness of the MWCNTs was estimated as (*D*_out_ − *D*_in_)/2 (*D*_out_: External diameter, *D*_in_: Internal diameter of MWCNTs). Morphometric analysis was performed using standardized pictures obtained on a magnification of 100,000×. Quantitative measurements of diameters of MWCNTs (*n* = 100 for each sample) on TEM images were carried out using the software Image-Pro Plus 6.0.0.260 (Media Cybernetics, Inc., Rockville, MD, USA). Statistical processing was carried out using STATISTICA 10 software (TIBCO Software Inc., Palo Alto, CA, USA). To compare independent samples, the Student’s *t*-test was used. The differences in the control and experimental samples were considered significant for *p* < 0.01.

### 2.5. Raman Spectroscopy

Raman measurements were performed at room temperature with a confocal Raman microscope NTEGRA Spectra (NT-MDT, Zelenograd, Russia). The spectra were recorded with an electron multiplying charge coupled device (Newton, Andor; Abingdon, Oxfordshire, UK) cooled down to −65 °C. The recorded spectra covered the range between 600 and 3000 cm^−1^ (diffraction grating with 600 lines per mm). For the measurements, a 532 nm laser was used; the intensity was adjusted to 2 mWt.

The samples were deposited on aluminum foil. On each sample, 15 random points were analyzed; the spectrum acquisition time was 100 s for each point. Thus, 15 spectra were collected for each sample. They were processed with median filtering (via Origin 8.6 software, OriginLab, Northampton, MA, USA) to reduce the noise; then the heights of the *D* and *G* peaks above the background were measured. Finally, the *I*_D_/*I*_G_ ratio was determined. To compare independent samples, the Student’s *t*-test was used.

### 2.6. Energy-Dispersive X-Ray Spectroscopy (EDS)

The microanalysis through EDS was performed with a detector (Oxford Instruments Inca X-Max 8 mm^2^; Abingdon, Oxfordshire, UK) coupled to the vacuum chamber of the analytical transmission electron microscope (JEM 2100, Tokyo, Japan) with Inca software (Oxford Instruments, Abingdon, Oxfordshire, UK). The obtained data was converted to Excel (Office 2016, Microsoft, Redmond, WA, USA) from its original format. Each sample was measured in 5–13 areas. To compare independent samples, the Mann-Whitney test was used.

## 3. Results and Discussion

### 3.1. Aqueous Suspension of MWCNTs

**Figure 1:**
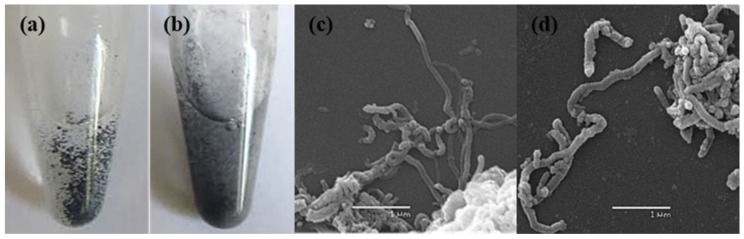
Suspensions and SEM images of MWCNT-t (**a**,**c**) and MWCNT-d (**b**,**d**). Pictures of MWCNT suspensions were taken immediately after sonication in water. The scale bar on the SEM images is 1 μm.

Both studied nanomaterials are loose black powders with weak electrostatic properties. Due to the hydrophobic nature, both types of MWCNTs aggregate and precipitate after their resuspension in water (it takes several minutes to sediment completely). MWCNT-t (Figure 1a) form larger aggregates in suspension and tend to sediment faster than MWCNT-d (Figure 1b). The latter is more dispersed in suspension.

### 3.2. Characterization of Intact MWCNTs by Scanning Electron Microscopy

SEM images of industrial MWCNTs demonstrate that nanotubes form aggregates up to a few microns in size. These aggregates can be present in a dry MWСNTs sample or form de novo in water. The nanotubes have a twisted shape; their length varies from 100 nm to several microns. The two types of MWCNTs differ in thickness; most MWCNT-t (Figure 1c) is, on average, two times thinner than the MWCNT-d (Figure 1d) that have thicknesses of about 80 nm.

### 3.3. Characterization of Hypochlorite-Induced Degradation of MWCNTs by Transmission Electron Microscopy (TEM)

**Figure 2:**
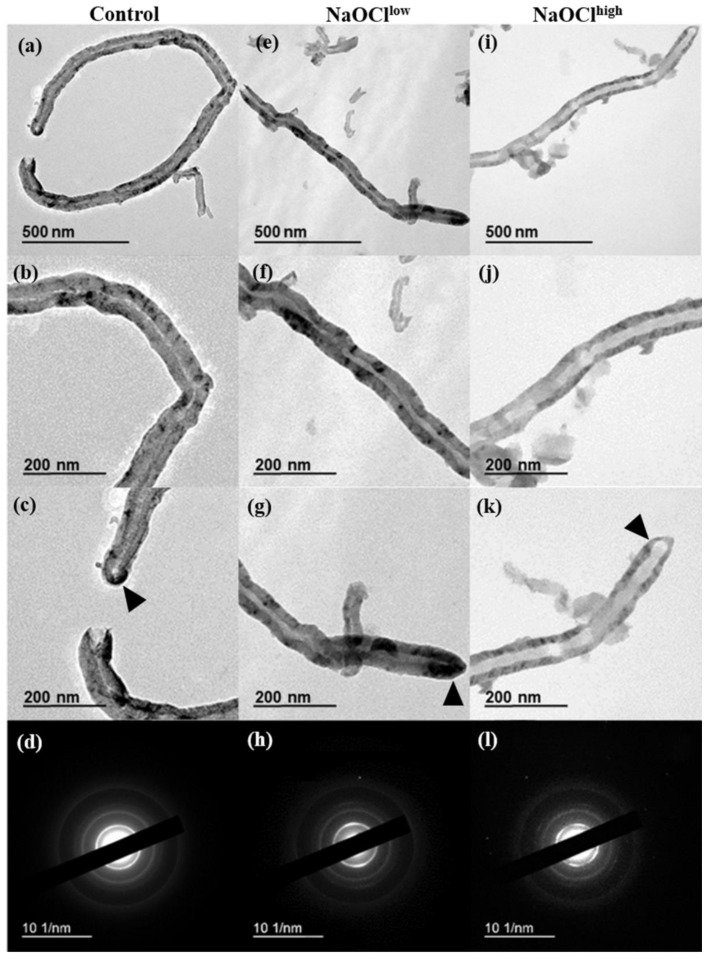
MWCNT-t: (**a**–**d**) intact nanotubes; (**e**–**h**) MWCNTs after several additions of NaOCl^low^; (**i**–**l**) MWCNTs after several additions of NaOCl^high^; (**a**,**e**,**i**) general view; (**b**,**f**,**j**) the central area of MWCNTs, the inner channel and the walls are clearly seen; (**c**,**g**,**k**) tips of nanotubes, rounded cap is retained (pointed by arrows); and (**d**,**h**,**l**) electron diffraction patterns.

**Figure 3:**
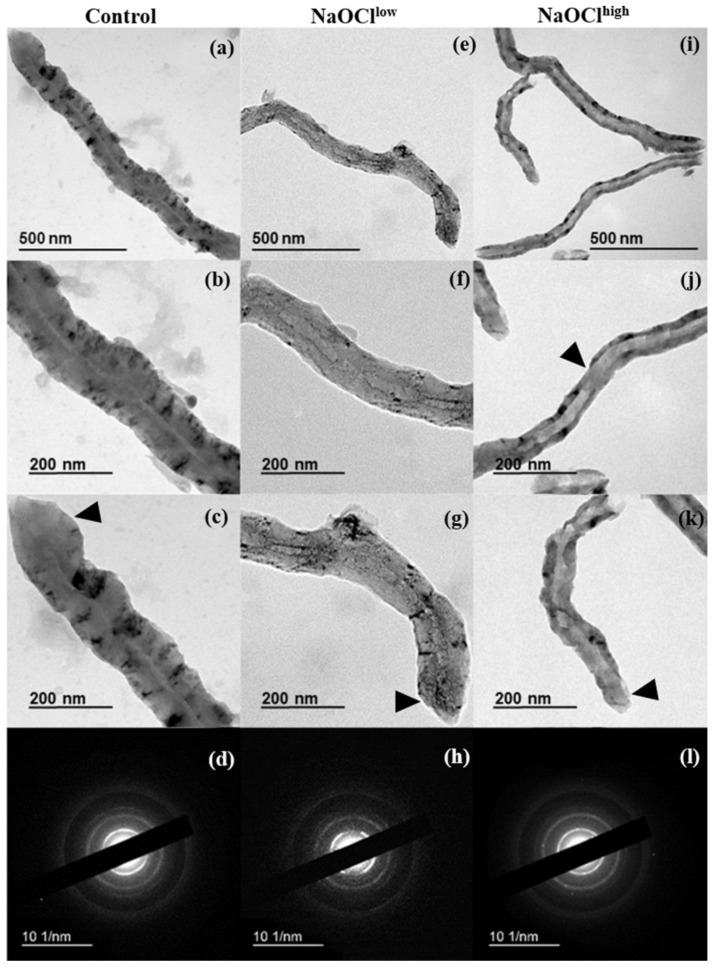
MWCNT-d: (**a**–**d**) intact nanotubes; (**e**–**h**) MWCNTs after several additions of NaOCl^low^; (**i**–**l**) MWCNTs after several additions of NaOCl^high^; (**a**,**e**,**i**) general view; (**b**,**f**,**j**) the central area of MWCNTs, the inner channel and the walls are clearly seen; (**j**) shows the wall thinning (pointed by arrow); (**c**,**g**,**k**) tips of nanotubes, rounded cap is retained, except (**k**) (pointed by arrows); and (**d**,**h**,**l**) electron diffraction patterns.

The TEM images showed that both nanomaterials share features which are typical for industrial-grade MWCNTs—the twisted shape, the presence of broken tips on one or both ends, the narrow internal channel (7–9 nm) (Figure 2a–c and Figure 3a–c ). Morphometric analysis of TEM images of control MWCNTs revealed that the average wall thickness was 18 ± 11 nm and 35 ± 12 nm for MWCNT-t and MWCNT-d, respectively (Table 1).

Treatment of MWCNTs by hypochlorite resulted in the reduction of their wall thickness. The average outer diameter (*D*_out_) of MWCNT-t did not change after NaOCl treatment in the range of experimental error, whereas *D*_out_ of MWCNT-d was reduced by 20% after their exposure to NaOCl^high^ as compared to the control (Table 1). Importantly, for both types of MWCNTs, the average inner diameter (*D*_in_) increased by approximately 1.5-fold after CNT treatment with low concentrations of hypochlorite (NaOCl^low^) and by 2–3.5-fold after their treatment with high concentrations of the reagent (NaOCl^high^), as compared to control samples (Figure 2b,f,j and Figure 3b,f,j; Supplementary Information, Table S1). Individual MWCNT-d with nearly complete wall vanishings were observed in TEM images (Figure 3j). One of the closed tips of MWCNT-d could remain unaffected by oxidation, this did not correlate with morphological changes of their side-walls (Figure 2c,g,k and Figure 3c,g,k ).

Thus, NaOCl^high^ damaged MWCNT-d walls from both the inside and the outside up to the complete destruction of some local areas in the graphitic backbone, while the degradation of MWCNT-t was detected only from the inside. At the same time, the electron diffraction patterns showed that MWCNTs retained their crystal structure in the course of degradation (Figure 2d,h,l and Figure 3d,h,l ).

**Figure 4:**
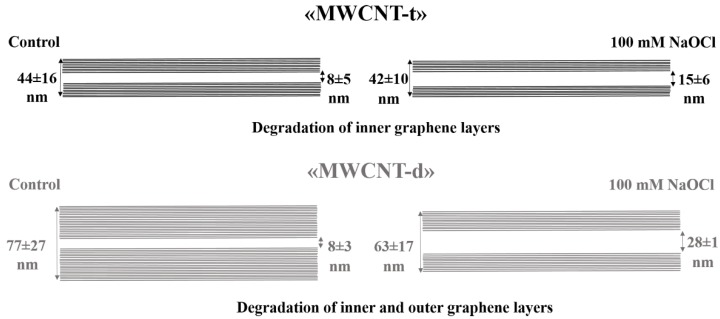
Scheme of MWCNT-t and MWCNT-d degradation under NaOCl^high^ treatment.

The distance between graphitic layers in MWCNTs varies within narrow limits and was reported to be 0.34 nm [30]. Based on this number, MWCNT-t consists of ≈53 graphene layers; MWCNT-d consists of ≈103 graphene layers. Thus, after incubation with NaOCl^high^, MWCNT-t lost ≈13 graphitic layers, and MWCNT-d lost ≈50 layers, which is 3.85 times higher (Figure 4). After NaOCl^low^ treatment, 6 and 9 layers were exfoliated from MWCNT-t and MWCNT-d, respectively.

Thus, the dynamics of degradation for MWCNT-t and MWCNT-d are not the same. The preferable sites for NaOCl oxidation also differ; destruction of MWCNT-d (thicker tubes) occurred both on the inside and the outside, whereas MWCNT-t was degraded only on the inner side. The rapid degradation of the inner layers of MWCNTs may be a consequence of both their high defectiveness and the overall tendency of the system to minimize its energy. The inner MWCNT graphitic layers have higher energy than outer ones; the energy, which is necessary to bend a graphitic monolayer into a cylinder of radius *R*, is proportional to 1/*R* [31].

The destruction of both inner and outer graphene layers was reported previously in the study of macrophage-induced biodegradation of MWCNTs [20,32]. Elgrabli et al. studied thick laboratory-grade MWCNTs (*D*_out_ = 120–140 nm) with a relatively wide inner channel (*D*_in_ = 40–80 nm). These nanotubes degraded by ROS produced upon electron-beam irradiation of MWCNT water suspensions or upon intracellular ROS generation by THP-1 cells.

According to some of the existing data, the concave surface of carbon nanotubes (inner layers of graphene) is less reactive than the convex (outer layers). It happens because when a graphene layer is bent, electron density shifts from the concave surface to the convex surface [33]. Thus, it is expected that MWCNTs should degrade from the convex side.

The obtained result is inconsistent with the previously reported direct proportional relation of MWCNT oxidative stability and their *D*_out_ [34]. MWCNT-t, due to the smaller *D*_out_, should be less stable. The observed degradation of MWCNT-d and higher stability of MWCNT-t evidence that not only the outer diameter, but also other structural characteristics, may determine the effectiveness of MWCNT degradation. We suggest that the reason for these inconsistencies is connected with the defectiveness of the MWCNTs. One may suppose that the amount and nature of structural defects are of great importance for their biodegradation [10,18].

### 3.4. Characterization of Hypochlorite-Induced Degradation of MWCNTs Using Raman Spectroscopy

Raman spectroscopy was employed to probe the defectiveness of MWCNTs and to characterize the degradation process. The Raman spectra of carbon materials contain two characteristic peaks. The G-peak at ~1580 cm^−1^ is typical for graphite (sp2-hybridized carbon) and the D-peak at ~1350 cm^−1^ arises due to the defects, including dopant atoms, and sp3-hybridised carbon, etc. The ratio of the intensities *I*_D_/*I*_G_ is often used to characterize the disorder in carbon materials.

**Figure 5:**
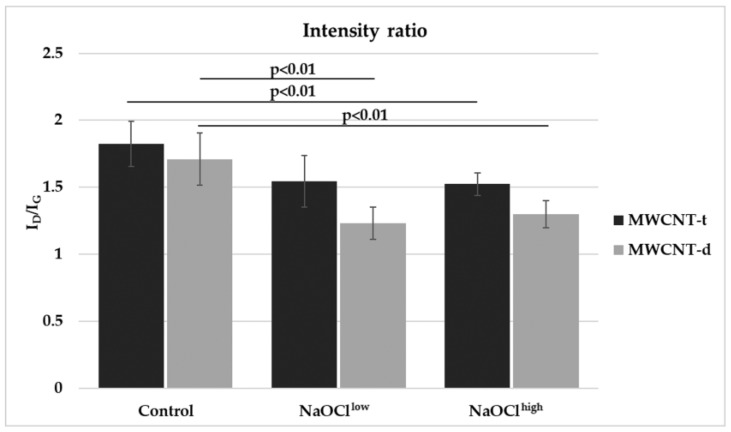
*I*_D_/*I*_G_ ratios of Raman spectra obtained for two samples of MWCNTs before and after NaOCl treatment. Statistically-significant differences are presented in Table S3 Supplementary Information.

We have shown that the average *I*_D_/*I*_G_ was 1.7 ± 0.2 and 1.8 ± 0.17 for MWCNT-d and MWCNT-t, respectively (Figure 5). Thus, both intact nanomaterials demonstrated a high defect density.

For experimental samples, a decrease in *I*_D_/*I*_G_ for MWCNT-d was significant both for NaOCl^high^ and NaOCl^low^ and equaled to 1.2 ± 0.12 and 1.3 ± 0.1, respectively. For MWCNT-t, the *I*_D_/*I*_G_ ratio was significantly reduced only when CNTs were exposed to NaOCl^high^ and reached values of 1.5 ± 0.1 (Figure 5). The decrease in *I*_D_/*I*_G_ indicated that the structure of MWCNTs had undergone changes and the defect density decreased by 20–30%.

With comparable initial defect density, MWCNT-d degraded, morphologically, more intensively, as evidenced by TEM. This may indicate the presence of defects differing in their chemical nature from those in MWCNT-t. The accessibility of defect sites for oxidants and their distribution between the layers within MWCNTs may also be a determining factor for MWCNTs degradation. The distribution of defects can differ in MWCNT graphitic layers depending on the synthesis conditions [34]. The most defect-free nanotubes are obtained by electric arc synthesis (without using a catalyst), while MWCNTs produced by the use of less expensive methods are characterized by higher defectiveness [17].

In our experiments, the peak height in the Raman spectra did not reduce after MWCNTs oxidation, which was observed during oxidation of graphene [35] or carboxylated SWCNTs [16]. Probably, in the present work, we observed the initial stage of CNT degradation when MWСNTs retain their tubular structure and do not transform into carbonaceous flakes, as happens after prolonged destructive impact [18]. As the carbon materials degrade, various changing patterns of *I*_D_/*I*_G_ ratio can be observed. If CNTs had a low defect density at the beginning of the process, the defectiveness increased during degradation. This was the case when lab-manufactured MWCNTs were degraded by the Fenton reagents [36] or by hypochlorite [19]. At the same time, the *I*_D_/*I*_G_ ratio may decrease during degradation of the initially defective material, since the most defective graphitic layers degrade first and cease to contribute to the spectrum [16].

From the TEM images, it was seen that MWCNT-d lost several layers that undoubtedly resulted in the exposure of the inner graphitic structures, which are less defective. In the range of experimental error, we did not detect the degradation of the outer graphitic lattice of MWCNT-t. Nevertheless, one cannot exclude the exfoliation of several outer layers of MWCNT-t after NaOCl treatment following exposure of inner graphitic structures.

### 3.5. Characterization of Hypochlorite-Induced Degradation of MWCNTs Using Energy-Dispersive X-Ray Spectroscopy

EDS was performed to compare the two types of MWCNTs chemically. EDS analysis showed that both carbon nanomaterials were qualitatively similar in their chemical composition. However, MWCNT-d had an impurity of aluminum (1 ± 0.3%). The percentage of oxygen in MWCNT-t and MWCNT-d was similar.

**Figure 6:**
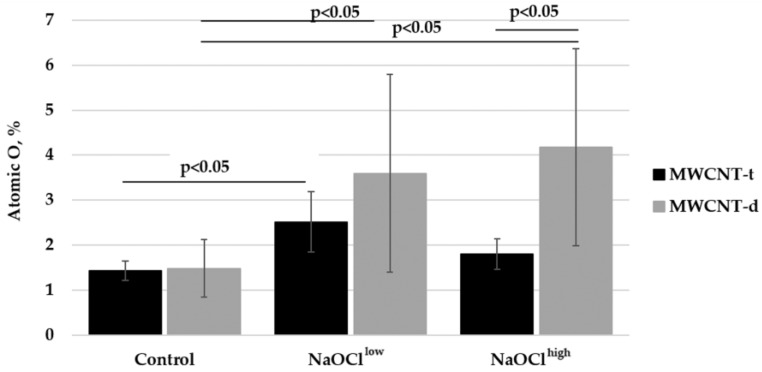
EDS analysis of oxygen content in two samples of MWCNTs before and after NaOCl treatment. Statistically-significant differences are presented in Table S4 Supplementary Information.

The amount of oxygen in MWCNT-t increased in a statistically significant way after NaOCl^low^ treatment (Figure 6). In the range of experimental error, NaOCl^high^-treated MWCNT-t had the level of oxygenation similar to that of the control sample. For MWCNT-d, both NaOCl^low^ and NaOCl^high^ treatment caused the statistically significant increases in oxygenation, as compared to the control sample. The relative amount of Al in MWCNT-d increased (3.6 ± 2.4% in NaOCl^high^-treated sample) due to MWCNT degradation and the decrease in carbon content.

The results presented in Figure 6 are consistent with the data obtained by TEM and Raman spectroscopy. After NaOCl^low^-treatment, morphometric analysis shown that MWCNT-d lost about 50% more graphene layers than MWCNT-t did (9 versus 6 layers) and this difference increased when MWCNTs were incubated with NaOCl^high^ (50 versus 13 layers). The better degradability of MWCNT-d may probably be explained by the difference in the type of initial defects and their accessibility for the oxidant in two types of MWCNTs.

Raman spectroscopy reveals nearly all existing MWCNT defects, such as vacancies, heptagon-pentagon pairs functional groups, and others [37]. Though MWCNT-t and MWCNT-d have similar *I*_D_/*I*_G_ and the level of oxygen, the exact chemical nature of the defects of the two types of MWCNTs is expected to differ due to the different synthesis conditions. Treatment with NaOCl caused a decrease in the total number of defects because of the degradation of the most defective outer layers of MWCNTs. At the same time, NaOCl increased the number of oxygen-containing functional groups through oxidation.

Higher oxygen content in MWCNTs indicates the presence of defects in the form of additional functional groups (hydroxyl, carbonyl, or carboxyl). Such defects may also appear during MWCNTs synthesis or purification [17]. It was shown that the treatment with strong oxidants such as HNO_3_, KMnO_4_, H_2_SO_4_/HNO_3_, (NH_4_)_2_S_2_O_8_, H_2_O_2_, and O_3_ increases the amount of these functional groups on the MWCNTs surface [38]. These functional groups are the main target for further oxidation.

Additionally, as the synthesis of CNTs frequently requires the presence of catalytic metals in the manufacturing process, CNTs may contain several residual metal impurities, the concentrations of which may be relatively high in industrial-grade CNTs [39]. The presence of metal impurities may influence the degradation of MWCNTs. For both the studied nanomaterials, nickel was used as a synthesis catalyst. MWCNT-d, in contrast to MWCNT-t, contained Al in addition to Ni. Aluminum is considered to be an impurity that may often be found in some quantity in industrial-grade MWCNTs [40]. In the pristine [as-produced] samples, residual materials include alumina as support substances (i.e., and silica). It was also shown that the presence of metal impurities may influence some properties of MWCNTs such as redox and electrostatic properties [40]. It is known that redox active metals associated with CNTs (e.g., iron) can induce oxidative stress and toxicity [41,42]. Iron was shown to be the inhibitor of MWCNT degradation in macrophages [32]. Metal impurities can induce oxidative stress and inflammation; the iron residues caused a remarkable decrease in the toxicity of MWCNTs [43]. It was shown that Au-nanoparticles increased MWCNT stability under electron beam irradiation [44]. However, there are no works that highlight the role of aluminum in CNT degradation. According to data on hypochlorite reactivity, it is able to interact with Al (http://www.forceflow.com/hypochlorite/SodiumHypoIncompatibilityChart.pdf), leading to a greater variability in chemical reactions. However, the influence of the impurities on the properties of MWCNTs is still poorly studied.

## 4. Conclusions

The physiological steady-state concentrations of sodium hypochlorite at the inflammatory site may reach a value of millimolar concentrations [12,13]. In the present work, we showed that degradation of MWCNTs in suspensions occurs upon their treatment with NaOCl that is added several times at concentrations of 1 mM. This implies that, after entering the body, industrial-grade MWCNTs may be slowly degraded in tissues through NaOCl-induced oxidation. The oxidation of MWCNTs’ surface increases their solubility and dispersion rate [45]. It was also shown that better dispersion decreases the cytotoxity of MWCNTs [46]. In the present work we have shown for the first time that MWCNTs degrade mostly from the inner side upon sodium hypochlorite treatment. Our study revealed that under identical incubation conditions (temperature, concentration of NaOCl, and duration of exposure), MWCNTs with thicker walls may degrade to a greater degree; the thickness is not the only factor affecting MWCNT degradation. The rate of degradation may depend on a variety of factors such as the synthesis conditions, the presence of impurities and the type of chemical defects. By regulating these factors, one may design nanotubes in a way that increases or decreases their degradation time, depending on the goals set. For industrial production, it may be important to synthesize more chemically stable MWCTNs with greater durability, as well as MWCNTs with a greater ability to degrade—in order to decrease their potential harm to the environment and human health [43]. The controllable propensity of MWCNTs for biodegradation may also be important for medical applications in targeted drug delivery, when it is necessary to regulate the drug carrier decay time.

## Figures and Tables

**Table 1:** Morphometric parameters of intact MWCNTs and MWCNTs treated with NaOCl.

Parameter	*D*_out_, nm		*D*_in_, nm		Avg. Wall Thickness, nm (%)	
Sample	MWCNT-t	MWCNT-d	MWCNT-t	MWCNT-d	MWCNT-t	MWCNT-d
Control	44 ± 16	77 ± 27	8 ± 5 *	8 ± 3 #	18 ± 11 (100%)	35 ± 12 (100%)
NaOCl^low^	44 ± 12	76 ± 22 $	12 ± 6 *	13 ± 3 #	16 ± 9 (89%)	32 ± 13 (91%)
NaOCl^high^	42 ± 10	63 ± 17 $	15 ± 6 *	28 ± 1 #	14 ± 8 (78%)	18 ± 9 (51%)

Data expressed as Mean values ± SD. Statistically-significant difference: *, $, # *p* < 0.01 presented in Table S2 of the Supplementary Information.

## Supplementary Materials

The following are available online at http://www.mdpi.com/2079-4991/8/9/715/s1, Table S1: Additions of NaOCl and water to suspensions of MWCNTs (0.5 mg/mL in water); Table S2: Morphometric analysis. Statistically-significant difference between experimental groups; Table S3: Raman spectroscopy. *I*_D_/*I*_G_ ratios of Raman spectra. Statistically-significant difference between experimental groups; Table S4: Energy-dispersive X-ray spectroscopy. Oxygen content. Statistically-significant difference between experimental groups.
