# Supplementary material for: Wall Thickness of Industrial Multi-Walled Carbon Nanotubes Is Not a Crucial Factor for Their Degradation by Sodium Hypochlorite
Source: Nanomaterials (Basel). 2018 Sep 12;8(9):715. doi: 10.3390/nano8090715 (PMC6164318; doi:10.3390/nano8090715)
Supplement: Supplementary file 1 [file nanomaterials-08-00715-s001.zip › nanomaterials-351837-SI.pdf]

nanomaterials

Supporting Information for

Wall Thickness of Industrial Multi-Walled Carbon Nanotubes Is Not a Crucial Factor for Their Degradation by Sodium Hypochlorite

Alexander G. Masyutin [1,*], Dmitry V. Bagrov [2], Irina I. Vlasova [2], Igor I. Nikishin [1], Dmitry V. Klinov [2], Ksenia A. Sychevskaya [3], Galina E. Onishchenko [1] and Maria V. Erokhina [1]

Table S1. Additions of NaOCl and water to suspensions of MWCNTs (0.5 mg/ml in water)

$NaOCl^{low}$ – low concentrations of NaOCl, stock solution was 100mM NaOCl, the concentration of NaOCl in a sample after addition of one unit dose was (1.0 ± 0.1) mM;

$NaOCl^{high}$ – high concentrations of NaOCl, stock solution was 1.2 M NaOCl, the concentration of NaOCl in a sample after addition of one unit dose was within the range 67–93 mM.

After NaOCl additions, the samples were mixed by the use of rotator Multi Bio RS-24 for 3 - 4 hours.

250 µl of MWCNTs in 0.5-ml eppendorfs

Day of additions	Time	Control	$NaOCl^{low}$<br>100 mM NaOCl, µL	$NaOCl^{high}$<br>1.2 M NaOCl, µL
**1**	3 pm		2.5	21
**2**	10 am<br>6 pm		2.5<br>2.5	21
**3**	10 am		2.5	
**4**	11 am		2.5	
**5**	6 pm		2.5	21
**6**		63 µL water	48 µL water	
**7**	11 am<br>6 pm		3<br>3	21
**8**	14 pm		3	21
**9**	12 pm		3	
**10**	10 am	63 µL water	3<br>21 µL water	21

Table S2. Morphometric analysis. Statistically-significant difference between experimental groups.

Statistical processing was carried out using STATISTICA 10 software. To compare independent samples, the Student's test was used. The differences in the control and experimental samples were considered significant for p <0.01.

Parameter/ Samples	MWCNT-t		MWCNT-d	
	D_{out}	D_{in}	D_{out}	D_{in}
$NaOCl^{low}$ versus Control	p>0,05	p=0,00005	p>0,05	p=0,00000
$NaOCl^{high}$ versus Control	p>0,05	p=0,00000	p=0,0002	p=0,00000
$NaOCl^{high}$ versus $NaOCl^{low}$	p>0,05	p=0,002	p=0,0001	p=0,00000

Table S3. Raman spectroscopy. I_D/I_G ratios of Raman spectra. Statistically-significant difference between experimental groups.

Statistical processing was carried out using STATISTICA 10 software. To compare independent samples, the Student's test was used. The differences in the control and experimental samples were considered significant for p <0.01.

I_D/I_G ratio/ Samples	MWCNT-t	MWCNT-d
$NaOCl^{low}$ versus Control	p=0,006	p=0,000005
$NaOCl^{high}$ versus Control	p=0,0032	p=0,005
$NaOCl^{high}$ versus $NaOCl^{low}$	p>0,05	p>0,05

Table S4. Energy-dispersive X-ray spectroscopy. Oxygen content. Statistically-significant difference between experimental groups.

Statistical processing was carried out using STATISTICA 10 software. To compare independent samples, the Mann-Whitney test was used. The differences in the control and experimental samples were considered significant for p <0.05.

Samples	MWCNT-t	MWCNT-d
$NaOCl^{low}$ versus Control	p=0.001	p=0.02
$NaOCl^{high}$ versus Control	p>0,05	p=0.015
$NaOCl^{high}$ versus $NaOCl^{low}$	p>0,05	p>0,05

© 2018 by the authors. Submitted for possible open access publication under the terms and conditions of the Creative Commons Attribution (CC BY) license (http://creativecommons.org/licenses/by/4.0/).
